# Spatiotemporal Risk of Bacillary Dysentery and Sensitivity to Meteorological Factors in Hunan Province, China

**DOI:** 10.3390/ijerph15010047

**Published:** 2017-12-29

**Authors:** Chengdong Xu, Gexin Xiao, Jinfeng Wang, Xiangxue Zhang, Jinjun Liang

**Affiliations:** 1 State Key Laboratory of Resources and Environmental Information System, Institute of Geographic Sciences and Natural Resources Research, Chinese Academy of Sciences, Beijing 100101, China; xucd@lreis.ac.cn (J.W.); zxx@lreis.ac.cn (X.Z.); 2 China National Center for Food Safety Risk Assessment, Beijing 100022, China; 3 Jiangsu Center for Collaborative Innovation in Geographical Information Resource Development and Application, Nanjing 210023, China; 4 The School of Earth Science and Resources, Chang’an University, Xi’an 710054, China; 5 Hunan Provincial Center for Disease Control and Prevention, Changsha 410005, China; LiangJinjun@126.com

**Keywords:** bacillary dysentery, spatial-temporal risk, meteorological factors, spatial-temporal model

## Abstract

Bacillary dysentery remains a public health concern in the world. Hunan Province is one of the provinces having the highest risk of bacillary dysentery in China, however, the spatial-temporal distribution, variation of bacillary dysentery and sensitivity to meteorological factors in there are unclear. In this paper, a Bayesian space-time hierarchical model (BSTHM) was used to detect space-time variation, and effects of meteorological factors between 2010 and 2015. The risk of bacillary dysentery showed apparent spatial-temporal heterogeneity. The highest risk occurred in the summer season. Economically undeveloped mountainous areas in the west and south of the province had the highest incidence rates. Twenty three (18.9%) and 20 (16.4%) counties were identified as hot and cold spots, respectively. Among the hotspots, 11 counties (47.8%) exhibited a rapidly decreasing trend, suggesting they may become low-risk areas in the future. Of the cold spot counties, six (30%) showed a slowly decreasing trend, and may have a higher risk in the future. Among meteorological factors, air temperature, relative humidity, and wind speed all played a significant role in the spatial-temporal distribution of bacillary dysentery risk. These findings can contribute to the implementation of an early warning system for controlling and preventing bacillary dysentery.

## 1. Introduction

Bacillary dysentery in humans mainly results from infection by different species of Shigella bacteria [1]. The disease is transmitted via the oral-fecal route or through contact with contaminated water and food. The incubation period is 1 to 4 days, and clinical symptoms of the disease include diarrhea, stomach cramps, and fever [2]. Bacillary dysentery remains a public health concern in both developed and developing countries. Worldwide, there are more than 160 million cases of shigellosis per year, resulting in about 1 million deaths [3]. In China, bacillary dysentery represents a considerable public health burden, and nearly 140,000 cases of bacillary and amebic dysentery occur on a yearly basis, placing this disease within the top five infectious diseases in the country in 2015 [4].

The risk of bacillary dysentery shows spatial heterogeneity, and the incidence rates of bacillary dysentery vary considerably worldwide. At a global scale, developing counties have a reportedly greater risk than developed counties. For example, Kotloff et al. estimated that about 164 million shigellosis episodes occurred worldwide over one year, of which 163 million were in developing countries [3]. In China, Zhang et al. reported that high-risk regions were mainly located in western China, where the level of economic development was relatively low [5]. Long-term stable factors in the local ecological environment, in addition to social-economic status, infrastructures, and sanitation conditions, are the main drivers of spatial patterns of disease risk.

The local and global risk of bacillary dysentery shows seasonality. Previous research demonstrated apparent seasonal variation in the incidence of bacillary dysentery in Shenyang and Jinan cities in northeastern and northern China, respectively, with bacillary dysentery infections apparently peaking in summer and fall seasons [6,7]. In Beijing City in northern China, peaks reportedly occurred from June to September [8]. In Vietnam, the highest incidence of shigellosis was reported from June to August [9]. In Dhaka City in southern Asia, the highest risk period extended from September to December [10]. Seasonality in the heterogeneous risk of bacillary dysentery indicates that climatic factors, such as air temperature, precipitation, relative humidity, wind speed, and sunshine hours, may play an important role in the varying temporal incidence of this disease [8,11,12,13,14].

Most previous studies have focused on the occurrence of bacillary dysentery and climatic factors in either time or space [7,15]. However, both spatial and temporal dimensions drive the incidence of bacillary dysentery. An understanding of spatial-temporal variance and the interacting factors that promote this disease would be helpful for controlling its incidence and spread.

The objectives of this study were: (1) to map the county-level spatial-temporal distribution and variation of bacillary dysentery risk; (2)to detect hot and cold spots and quantify their changes in disease risk; and (3) to explore the association of meteorological factors with bacillary dysentery.

## 2. Methods

### 2.1. Study Region and Data Source

Hunan Province is located in the middle reaches of the Yangtze River in central China and has a total population of about 67.83 million over an area of 211,800 square kilometers. The province contains many mountains and hilly landforms, which account for 66.62% of the total area. Hunan has a continental subtropical monsoon humid climate, with four distinctive seasons: a cold winter season, hot summer season, spring season with variable temperatures, and fall season in which temperatures drop steeply. Rains are concentrated in the spring and summer. Accordingly, this province experiences large intra-annual variation in its weather. The annual average temperature and rainfall among counties range from 16–19 °C and 1200–1700 mm, respectively. Although the flat area of the eastern belt has undergone industrial development, the economy of the western mountainous area remains relatively underdeveloped. A previous study indicated that Hunan Province had more outbreaks of bacillary dysentery than any other area in China [16].

### 2.2. Data Collection

**Figure 1:**
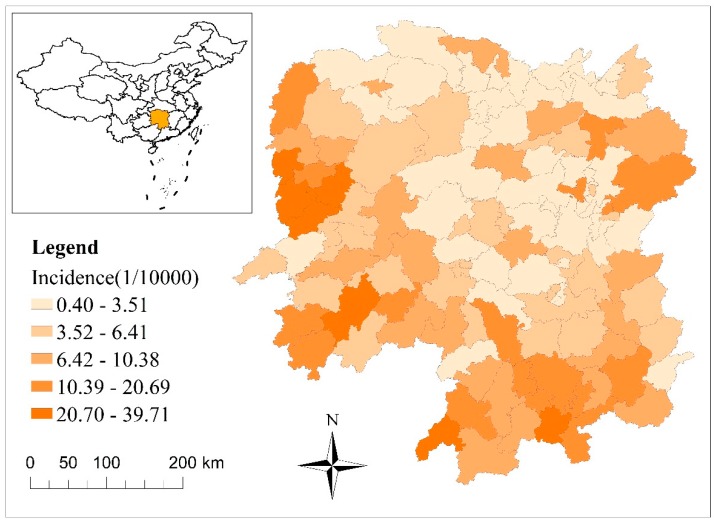
Cumulative incidence of bacterial dysentery in Hunan Province from 2010 to 2015.

**Figure 2:**
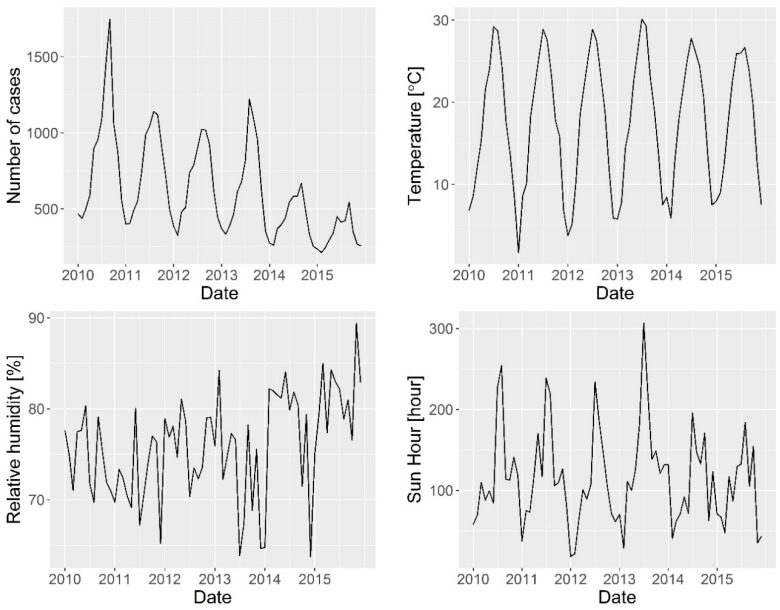

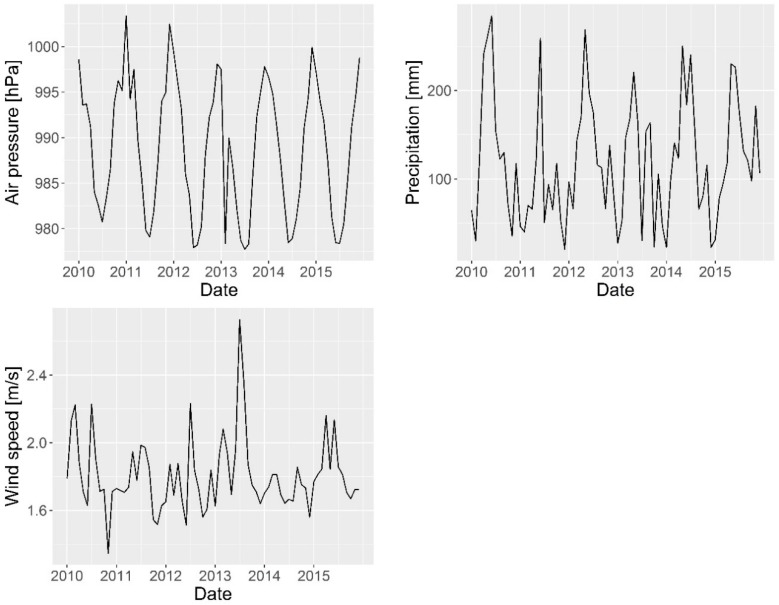
Temporal variation in meteorological factors from 2010 to 2015.

The study period was 1 January 2010 to 31 December 2015. Data on monthly cases of bacillary dysentery was obtained from the Centre for Disease Control and Prevention of Hunan Province (Figure 1). Monthly meteorological data in the same period were obtained from China’s Meteorological Data Sharing Service System (Figure 2) and included air temperature, precipitation, relative humidity, air pressure, wind speed, and hours of sunshine. The collinearity between these meteorological variables was tested. The highest Pearson’s correlation between the variables was 0.71, showing that there was no significant linear relation between the variables.

### 2.3. Bayesian Space-Time Hierarchy Model (BSTHM)

A BSTHM was used to analyze temporal and spatial heterogeneity in patterns of disease risk and variations in local trends [17], as well as spatial-temporal interactions in the occurrence of bacillary dysentery. The BSTHM can synthesize limited data, and use the prior probability distribution of unknown parameters to obtain the posterior probability of such parameters. Thus, to some extent, it can overcome problems in sample bias and ensure greater accuracy of the results. The BSTHM is more informative than traditional models. The model has the potential to reveal spatial-temporal local risks, characterize temporal variations, and detect risk factors for bacillary dysentery.

Poisson and log link regression functions were used to model the disease data. Assuming that *y_it_* and *n_it_* were the number of cases and the total population, respectively, in region *i* (*i* = 1, 2, …, 122) and month *t* (*t* = 1, 2, …, 72), the number of cases can be described as follows:

$$y_{it}∼Poisson(n_{it}r_{it})$$

where *r_it_* represented the relative risk of bacillary dysentery in region *i* and month *t*, which is expressed by the formula:

(1) $$\log(r_{it})=\alpha +s_{i}+(b_{0}t^{*}+v_{t})+b_{1i}t^{*}+\sum_{n=1}^{N}\beta^{n}x_{nit}+\varepsilon_{it}$$

where *α* was the overall log risk average for the entire study area (Hunan Province) in the designated period. In this model, the observed space-time heterogeneity in disease risk was divided into the following components. The spatial term *s_i_*, which was the ratio of the risk at *i* to the overall risk across Hunan Province, represented long-term stable factors, such as unsafe water, poor sanitation, an undeveloped economy, and topography. Meanwhile, (*b*_0_t* + *v_t_*) described the overall temporal trend common to all counties, wherein *b*_0_t* represented a linear component of the overall time trend, and *b_0_* was a regression coefficient. t* = *t* − 3.5 was a constant and indicated the middle of the study period. *v_t_* was additional Gaussian noise that represented a time random effect, which was usually caused by nonlinear variation. The combination of common spatial pattern and time trends represented the stable overall temporal-spatial component of disease risk. The term *b*_1*i*_ represented local trends of region *i* and quantified the deviation from the overall temporal variation (*b*_0_t* + *v_t_*). For example, if *b*_1*i*_ was larger than zero, this suggested that region *i* showed a faster decrease trend compared with the overall trend. If *b*_1*i*_ was smaller than zero, this suggested that region *i* had a weaker decrease trend. If *b*_1*i*_ was equal to zero, this indicated that region *i* had the same temporal trend as the overall trend. The space-time interaction term (*b*_1*i*_t* + *ε_it_*) denoted that differences existed in local time trends. *β* was the regression coefficient of the risk factors for the explanatory variable. *x_nit_* was the *n*-th risk factor in *i*-th region and *t*-th month. The term *ε*_1*i*_ denoted a random error term that included all the factors that were not considered in the model but affected the explanatory variable, which was assumed to follow a normal distribution. Prior distributions were assigned to all parameters. The spatial component *s_i_*, as well as the local trend coefficient *b*_1*i*_, was calculated by the Besag, York, and Mollie spatial model [18], in which spatially structured random effects and unstructured random effects were both considered. In the model, the spatial structure was expressed by a prior conditional autoregressive model (CAR), and unstructured random effects followed a Gaussian distribution. The conditional autoregressive prior was used to impose a spatial structure. The CAR prior on the spatial random effect implied that adjacent counties tended to have similar overall disease risks, which with a spatial adjacency matrix W, where diagonal entries *w_ii_* = 0 and off-diagonal entries *w_ij_* = 1 if areas *i* and *j* shared a common boundary and *w_ij_* = 0 otherwise. The temporal noise *v_t_* was modeled as *v_t_* ~ N (0, *σ_v_*^2^). The overdispersion parameter *ε_it_* was modeled as *ε_it_* ~ N (0, *σ*_ε_^2^). A Gaussian prior N (0, 0.1) was assigned to all random effect standard deviations (e.g., *σ_v_* and *σ_ε_*). All the regression coefficients *β* and *b*_0_, *b*_1_ also modeled as N (0, *σ*^2^) with the Gaussian prior of N (0, 0.1).

Based on the estimated posterior parameters of the BSTHM, the spatial-temporal heterogeneity and variance in bacillary dysentery risk in Hunan Province were quantified. All counties were classified as hot or cold spots representing higher or lower relative risks, respectively, as compared with the average risk in the entire province. The calculation process included two steps [19]. In the first step, a region was defined as a hotspot if the posterior probability $p\left(\exp\left(s_{i}\right)>1|\mathrm{data}\right)$ was greater than 0.95, whereas a region was regarded as a cold spot if the posterior probability $p\left(\exp\left(s_{i}\right)>1|\mathrm{data}\right)$ was less than 0.05. The remaining regions were regarded as neither hot nor cold spots. Here, exp(*s_i_*) represented the average disease risk (over time) in county *i* relative to *α* [20]. In the second step, the regions belonging to each risk category according to the above step were classified into different trend types based on the posterior probability of local slope *b*_1*i*_. If $p\left(b_{1i}>0|h_{i},\;data\right)>0.95$, a faster increasing/decreasing trend was assumed to be presented, as compared with the overall trend. If $p\left(b_{1i}>0|h_{i},\;data\right)<0.05$, a slower increasing/decreasing trend was assumed to be presented. Finally, if $0.05\leq p\left(b_{1i}>0|h_{i},\;data\right)\leq 0.95$, the trend was no different from the overall trend. In this way, the entire study area was classified into nine categories (three risk categories × three trend categories).

All the above parameters were estimated using data on numbers of cases, populations, and metrological factors. The model was implemented in the WinBUGS statistical software designed specifically for Bayesian research [21]. The posterior probability mean of all the parameters in the model was estimated through Markov chain Monte Carlo simulations.

## 3. Results

### 3.1. Spatiotemporal Patterns

Between 1 January 2010 and 31 December 2015, in total, 44,926 bacillary dysentery cases were reported in Hunan Province. The largest number of cases recorded in 2010, with an annual incidence was 1.58 out of every 10^4^ people. The smallest number of cases recorded in 2015, with an annual incidence was 0.60 out of every 10^4^ people.

There was an obvious seasonal pattern in the incidence rates of bacillary dysentery throughout the year. In particular, cases peaked during the summer season (May, June, and July), with an average monthly incidence was 0.65 out of every 10^4^ people. During the winter season (December, January, and February), the average monthly incidence was 0.32 out of every 10^4^ people (Figure 2).

**Figure 3:**
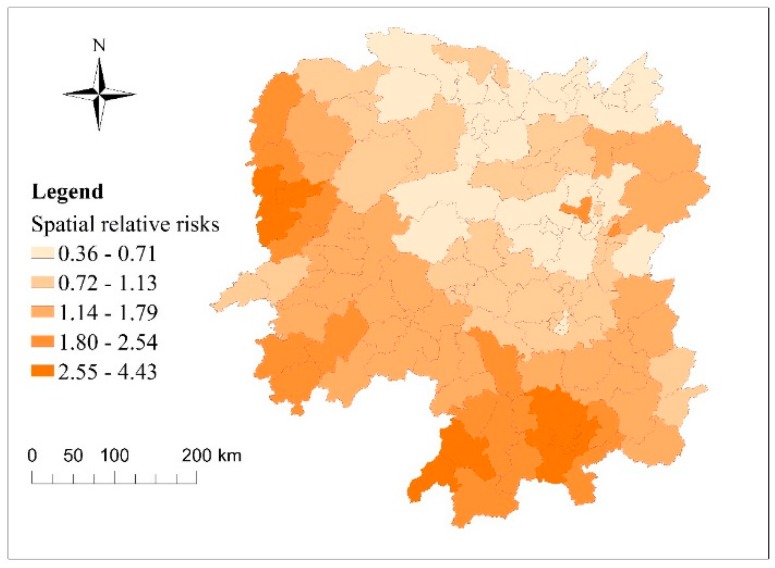
Spatial relative risks (RRs) (exp(*s_i_*)) of bacillary dysentery in counties in Hunan Province.

Figure 3 depicts the spatial relative risks (RRs) of bacillary dysentery in the study region from 2010 to 2015. The results suggested that the distribution of bacillary dysentery risk was spatially heterogeneous. The spatial RR values in counties in western and southern mountainous areas of the study region were higher (>1) than those of counties in other parts of the study region, implying that these regions had a relatively greater risk of bacillary dysentery. The highest level of stable spatial RR (>2) was in Wuling, a mountainous area in the western region, with the Nanling, a mountain to the south. Conversely, counties in eastern and northern flat regions had a lower level of bacillary dysentery risk and a spatial RR < 1.

**Figure 4:**
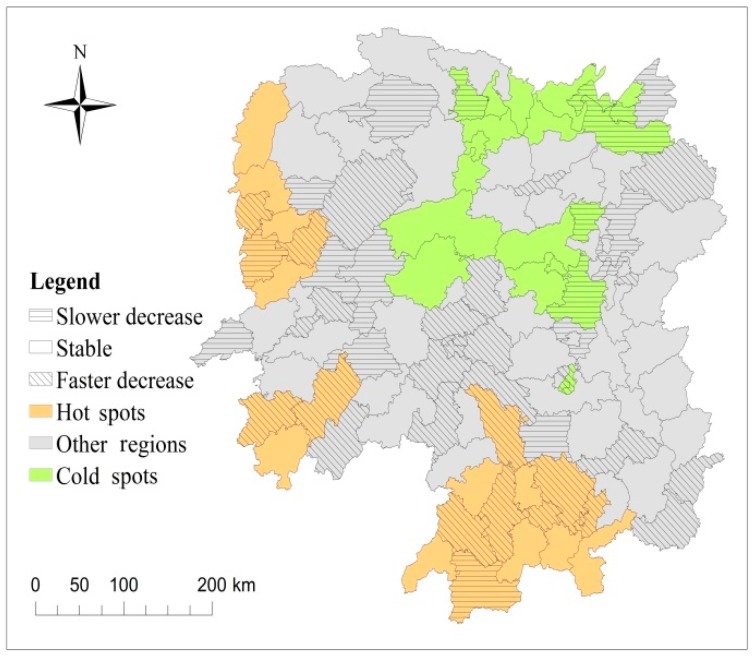
Spatial relative risks (RRs) (exp(*s_i_*)) and deviations in local trends compared to the overall trend *b*_1*i*_ of bacillary dysentery in each county of Hunan Province.

As shown in Table 1, in the study region, 23 (18.9%) and 20 (16.4%) counties were classified as hot and cold spots, respectively. Another 79 (64.7%) counties were identified as neither hot nor cold spots. As observed in Figure 4, hotspot regions were mainly distributed in economically underdeveloped mountainous areas in western and southern regions of the province.

**Figure 5:**
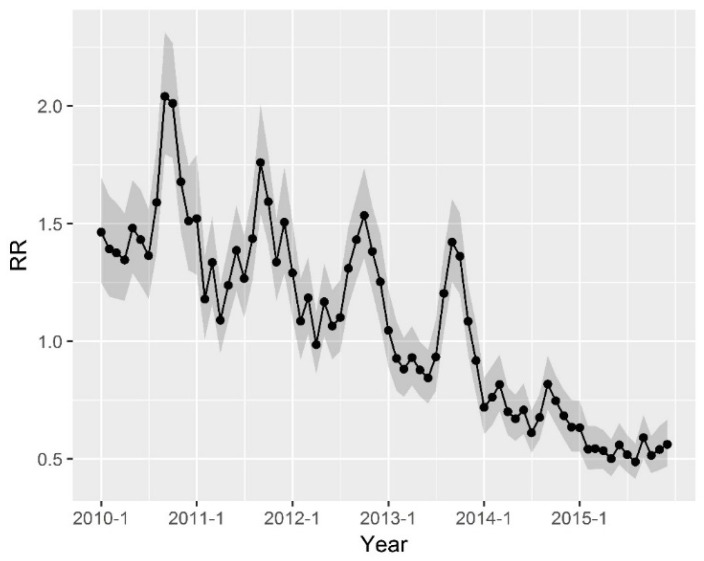
Overall temporal trend (exp(*b*_0_t*+ *v_t_*)) of bacillary dysentery in Hunan province (the grey shade is confidence interval).

Figure 5 shows the overall temporal trend of bacillary dysentery risk from 2010 to 2015. As can be seen, there was an average decreasing trend of 1.68% per year. Although the overall trend showed a decrease, the incidence of the disease showed large seasonal variation. The highest disease risk occurred in summer and fall seasons, and the lowest disease risk occurred in winter and spring.

The temporal trends varied per county. As presented in Table 1, the temporal trend in 11 (47.8%) of 23 hot spot counties showed a rapidly decreasing trend as compared with the overall trend. These counties will likely have a lower risk or even cease to be hot spots in the future. In contrast, two (8.7%) of 23 hot spot counties showed a slowly decreasing trend in comparison with the overall trend. Attention should be paid to disease control and prevention in these areas, as they will likely continue experience a greater RR in the future. Finally, the temporal trend in 10 (43.5%) of 23 hot spot counties was consistent with the overall trend.

Regarding 20 cold spot counties, two (10%) exhibited a faster decreasing trend than the overall decreasing trend. Thus, these countries will likely have a low risk and continue to be cold spots in the future. Furthermore, six (30%) of the 20 cold spots counties showed a slower decreasing trend than the overall trend. Therefore, the risk in these counties will likely be greater than the overall risk in the future, as compared with other areas in the region. Finally, the trend in 12 (60%) of the 20 cold spot counties was consistent with the overall trend. Thus, the current risk level in these counties will be maintained in the future.

Of the 79 counties that were neither hot nor cold spots, 17 (21.5%) showed a faster local decreasing trend than the overall decreasing trend, indicating that these counties will likely become cold spots in the future. In addition, 12 (15.2%) of the 79 counties that were neither hot nor cold spots showed a slower decreasing trend as compared with the overall decreasing trend. Therefore, these counties will probably have a higher RR than the overall risk of other counties in the future. The public health department should focus its attention on these counties. Finally, the trend in 50 (63.3%) of the 79 counties that were neither hot nor cold spots was consistent with the overall trend.

### 3.2. Risk Factors Analysis

A positive association was found between mean temperature and bacillary dysentery. An increase of 3.194% (95% confidence interval CI: 2.361 to 4.057) in the incidence of bacillary dysentery occurred with a rise in temperature of 1 °C (RR: 1.032; 95% CI: 1.024 to 1.041) (Table 2).

There was also a positive association between bacillary dysentery and relative humidity. A 1% increment in relative humidity was associated with a 0.674% increase (95% CI: 0.094 to 1.274) in the incidence of bacillary dysentery (RR: 1.007; 95% CI: 1.001 to 1.013) (Table 2).

Air pressure and wind speed showed negative correlations with bacillary dysentery. A 1 hPa rise in air pressure and a 1 m/s increase in wind speed were associated with a 0.232% (95% CI: −0.293 to −0.166) and 18.540% (95% CI: −25.640 to −11.530) decrease, respectively, in the incidence of bacillary dysentery. The corresponding RRs were 0.998 (95% CI: 0.997 to 0.998) and 0.831 (95% CI: 0.774 to 0.891), respectively (Table 2).

In addition, precipitation and hours of sunshine showed a weakly positive relationship with bacillary dysentery. A 1 mm increment in precipitation and a 1 h increase in sunshine hours were associated with rises of 0.0031% (95% CI: −0.0277 to 0.0337) and 0.147% (95% CI: 0.050 to 0.245) in the number of bacillary dysentery cases, respectively. The corresponding RRs were 1.0000(95% CI: 0.9997 to 1.0003) and 1.001 (95% CI: 1.001 to 1.002), respectively. The estimated coefficient for precipitation was not statistically significant, as the parameter’s posterior distribution included 0 (Table 2).

## 4. Discussion

The incidence of bacillary dysentery has increased worldwide in recent years due to the existence of various risk factors, such as unsafe water, poor sanitation, undeveloped economies, and harsh environments [22]. In this study, a novel BSTHM was used to explore spatial-temporal patterns in the incidence of bacillary dysentery and to examine the association between bacillary dysentery and meteorological factors in Hunan Province in southern China. The risk of bacillary dysentery was relatively higher in western and southern areas of the province, and meteorological factors appeared to play an important role in disease risk.

In Hunan Province, the spatial distribution of bacillary dysentery risk was non-homogeneous. Areas with the highest incidence (hot spots) of the disease were mainly concentrated in the western and southern regions of the province in remote, mountainous regions, far from large cities, with undeveloped economies, poor living environments, and unhygienic conditions. The low level of economic development in these regions, as well as a lack of sufficient health and medical equipment, low education levels, unsafe water, and poor environmental conditions, may explain the high incidence of bacillary dysentery. This finding was consistent with that of previous studies [5,23,24]. The present study showed that the rate of decline in bacillary dysentery in about half the hot spot counties was faster than the average rate of decline. Thus may be the result of public health department attention in these areas.

In addition to being spatially non-homogeneous, the risk of bacillary dysentery exhibited apparent seasonal variation. During the entire study period, the highest incidence occurred in summer (May, June, and July), and the lowest incidence occurred in winter (December, January, and February). These results are mostly in agreement with those of other study in Changsha City of China [12]. However, a seasonal peak in Beijing City was observed from June to September [8], which one month later than that observed in Hunan Province. This discrepancy might be due to differences in the spatial scale of the data analyzed in these two studies. The seasonality of bacillary dysentery risk also indicated that climate factors likely played an important role in the temporal variance of this disease [8,11,12,13,14].

Many previous studies indicated that temperature affected the transmission of bacillary dysentery [11,12,13,14]. The present study found that temperature was positively associated with the risk of bacillary dysentery, with a rise of 1 °C in air temperature corresponding to a 3.194% increase in the risk of disease. This finding was consistent with that of previous reports. According to one study, a rise of 1 °C in air temperature led to a nearly 11% increase in the risk of the disease in Jinan City in eastern China [7] and a nearly 16% increase in Baoan in Shenzhen City, southern China [14]. In Peru, each 1 °C rise in air temperature was associated with an increase of about 8% in bacillary dysentery risk [25]. In the UK, a rise of 1 °C in temperature increased the risk of bacillary dysentery by nearly 5% [26]. Elevated temperatures may lead to increased exposure to pathogens, promote the growth of the bacteria, and prolong the survival of bacteria in the environment and contaminated food [25]. In addition, high temperatures may be associated with specific behavioral patterns in the population, such as increased demands for water or a conscious reduction of activities, which could accelerate the transmission of bacillary dysentery.

In the present study, relative humidity and precipitation showed a positive correlation with bacillary dysentery. Some previous studies reported similar results. For example, a study in northeastern China observed that bacillary dysentery was positively associated with relative humidity and precipitation [27], and a study conducted in Beijing showed that relative humidity and precipitation had a positive effect on bacillary dysentery [8]. Similar findings were reported in studies conducted in the Pacific Islands [28], and U.S. [29]. Conversely, a study conducted in Wuhan indicated that precipitation had a negative effect on the occurrence of dysentery [13]. In two cities in northern and southern China, Jinan and Baoan, a time-series regression model pointed to a lack of a strong association between precipitation and the incidence of dysentery [14]. Previous studies reported that droplets were a major carrier of intestinal infection-related viruses [30]. Increased contract rates with droplets on days with high rainfall and relative humidity may be a potential mechanism underlying the association of humidity and precipitation with bacillary dysentery.

Other meteorological factors considered in the present study also influenced the transmission of bacillary dysentery. For example, bacillary dysentery showed a negative correlation with air pressure and wind speed. A previous study indicated that air pressure affected the immune system [31]. High wind speeds may lead to increased evaporation, and viruses cannot survive and reproduce as easily in dry food and environment. High wind speeds will also decrease contact among people and is thus not conducive to the spread of bacillary dysentery.

In the present study, an increase in the number of hours of sunshine had a weakly positive relationship with bacillary dysentery. The potential mechanism may be that sunshine is associated with a prolonged rise in temperature, thus promoting the spread of the disease. A similar conclusion was found in a study conducted in Denmark [32]. However, other studies conducted in China, one in Chaoyang City [33] and the other in Beijing City [8], pointed to an inverse association between hours of sunshine and bacillary dysentery. Differences among the studies might be due to the use of diverse observation scales or regional characteristics, as different study areas may have different climatic conditions that influence the epidemiology of dysentery.

The present study has some limitations. In the model, we used data at the spatial scale of county and the temporal scale of month in the analysis, possibly introducing an ecological fallacy [34]. The use of more detailed geographic units (e.g., township) might have provided a more precise estimate of local disease risk. Short-term effects of meteorological factors could also have been quantified by the use of shorter time scales for the meteorological variables. Furthermore, other risk factors for bacillary dysentery that were not included in the model may have introduced some uncertainties.

## 5. Conclusions

The findings of the present study indicate that the spatial-temporal distribution of bacillary dysentery risk in Hunan Province in China is non-homogeneous, with hotspots mainly concentrated in the economically undeveloped mountainous areas in western and southern regions, and the temporal variation in disease risk shows spatial diversity. In addition, the risk of bacillary dysentery is highest during hot and humid weather. This study sheds light on the spatial-temporal pattern and variation of bacillary dysentery in this region. The findings can benefit decision making by public health departments concerning prevention and control of the disease.

## Figures and Tables

**Table 1:** Cross-classification of bacillary dysentery risk in the 122 counties of Hunan province.

	Faster Decrease Trend	Slower Decreasing Trend	Not Different from Common Trend	Total
Hot spots	11	2	10	23 (18.9%)
Cold spots	2	6	12	20 (16.4%)
Neither hot or cold spots	17	12	50	79 (64.7%)

**Table 2:** The estimated posterior means and RR of all coefficients.

Risk Factors	Posterior Mean (95% CI) (100%)	RR (95% CI)
Average temperature (°C)	3.194 (2.361–4.057)	1.032 (1.024–1.041)
Relative humidity (%)	0.674 (0.094–1.274)	1.007 (1.001–1.013)
Air pressure (h Pa)	−0.232 (−0.293–−0.166)	0.998 (0.997–0.998)
Sunshine hours (h)	0.147 (0.050–0.245)	1.001 (1.001–1.002)
Precipitation (mm)	0.0031 (−0.0277–0.0337)	1.0000 (0.9997–1.0003)
Wind speed (m/s)	−18.540 (−25.640–−11.530)	0.831 (0.774–0.891)

Note: Posterior Mean of parameter is calculated by the BSTHM method. RR, relative risk, which is an exponential transformation of the regression coefficients (posterior means).
